# Optical technologies in monitoring mobility and delivery of drugs and metabolic agents^1,☆^

**DOI:** 10.1016/j.addr.2025.115699

**Published:** 2025-09-26

**Authors:** Valery V. Tuchin, Tianhong Dai, Luís M. Oliveira

**Affiliations:** a Institute of Physics and Science Medical Center, Saratov State University, Saratov 410012, Russia; b Laboratory of Biophotonics, Tomsk State University, Tomsk 634050, Russia; c Institute of Precision Mechanics and Control, FRC “Saratov Scientific Centre of the RAS”, Saratov 410028, Russia; d Wellman Center for Photomedicine, Massachusetts General Hospital, Harvard Medical School, 15, Boston, USA; e *Physics Department, Polytechnic Institute of Porto* – *School of Engineering, Rua Dr. António Bernardino de Almeida 471, 4200-072 Porto, Portugal*; f Center of Telecommunications and Multimedia, Institute for Systems and Computer Engineering, Technology and Science, Campus da FEUP, Rua Dr. Roberto Frias, 4200-465 Porto, Portugal

Monitoring the mobility and delivery of drugs and metabolic agents in tissues and cells is a present concern in various areas such as clinical diagnostic and treatment, cosmetics, cryopreservation and food industry. Due to the interest in studying such phenomenon, various techniques have been used for *ex vivo* monitoring of drug mobility and delivery over the years. In the last two decades and in the context of studying the tissue immersion clearing method, the use of different spectroscopic and imaging modalities has produced innovative results related to the monitoring of drug and metabolic kinetics and their delivery to tissues and cells. With the results obtained in these studies, considering their high precision and possibility to perform *in vivo* evaluation, optical technologies have gained significant relevance to be used in many other applications where the monitoring of agent mobility and delivery is the major objective. As an introduction to the Special Issue of Advanced Drug Delivery Reviews (ADDR), with the title “Optical technologies in monitoring mobility and delivery of drugs and metabolic agents”, this article aims to present a comprehensive overview of the latest developments presented in the various papers included in this edition, and to discuss the current challenges and future opportunities for research and development in this topic. Current optical technologies used for monitoring of molecular mobility and drug delivery, typical particles and agents, and delivery methods discussed in this issue are presented in Fig. 1.

The mobility and delivery of drugs and metabolic products in human and animal tissues and cells is a field of research with major interest for diagnostic and treatment applications, including cancer and diabetes mellitus and many other living important diseases, cryopreservation of organs, tissue poisoning, dermatology and cosmetology. The optical monitoring of mobility and delivery of exogenous and endogenous metabolic products has evolved greatly in the last 10 years, and provides various advantages, such as the discrimination of the diffusion properties in normal and diseased tissues, possibility of noninvasive monitoring, characterization and optimization of various treatments, cosmetic or cryopreservation procedures and quantification of water bounding states in tissues. Considering food processing and preservation, the evaluation of sugar delivery in fruits and the dosage optimization of cryopreservation agents in other consumable products has been a topic of recent interest, where the application of optical methods to monitor the mobility and delivery of such agents in food products has also proven valuable for the food industry.

Both spectroscopic and imaging methods have recently taken advantage of the valuable properties of light to advance the research related to the monitoring of mobility and delivery of various products in human and animal tissues, leading to significant and innovative results as described in the various papers in this Special Issue of ADDR. The following sections provide a summarized but detailed description of these latest achievements, as well as point out the emerging challenges and future research directions that are required for the final application of light technologies for *in vivo* efficient monitoring of the mobility and delivery of drugs and metabolic products.

Spectroscopic methods have been used for various years in different fields of Biophotonics. Different spectroscopic modalities, such as optical, ultraviolet (UV), infrared, Raman or terahertz (THz), were used to evaluate the optical properties of tissues, tissue fluids and other biological components [1], but have also been used to monitor the mobility and delivery of optical clearing agents (OCAs) in tissues during optical clearing treatments, as described in the review by Oliveira et al. [2]. This paper discusses the recent developments in using kinetic spectroscopy and thickness measurements to evaluate the diffusion properties of OCAs in human and animal tissues. The use of such method in studies performed in the last 15 years allowed to estimate the characteristic diffusion time and diffusion coefficient of various OCAs in different tissues, discriminate between those diffusion properties in healthy and diseased tissues and observe a 5 % increase in the mobile water content from the healthy to the cancerous colorectal and kidney tissues. Due to the sensitivity of kinetic spectroscopy measurements in the visible range to such data that can be used for diagnosis, this method has proven valuable to be considered as a complementary diagnostic tool when analyzing biopsied tissues.

Other spectroscopy techniques, such as the Raman spectroscopy and the electron paramagnetic resonance (EPR) spectroscopy have also been used to monitor dermal penetration of drugs *via* hair follicle (HF) routes, as described in the review by Svenskaya et al. [3]. Due to the complex structure of the skin, the topical application of drugs, ointments and creams is most ineffective for treatment purposes. In similarity to other methods, such as the use of microneedles to deliver drugs directly beneath the stratum corneum, the selection of the HF routes for drug delivery to numerous cell populations within the skin and to the blood stream has gained recent research interest. As discussed in this paper [3], although there is still a reduced number of publications related to the use of EPR spectroscopy to monitor drug delivery through the HF, the use of Raman spectroscopy is most effective, since it allows to discriminate between some components that were delivered through this route in human and porcine skin, using their characteristic spectra.

A brief description of the various spectroscopic and imaging techniques that are commonly used to monitor the kinetics of OCAs during optical clearing treatments is made in the paper by Yu et al. [4]. This paper also discusses the strategies of free diffusion and external forces-driven advection, or alternative methods to enhance tissue permeability, to optimize OCA delivery for deeper tissue clearing, and how the different optical monitoring modalities are used in each case.

Considering drug delivery using gold nanoparticles (GNPs), the review of Dykman et al. discusses various studies, where surface-enhanced Raman spectroscopy (SERS), Fourier transform infrared spectroscopy (FTIR) and fluorescence spectroscopy were used to monitor drug release and biodistribution [5]. According to the authors, GNPs have exceptional size and surface characteristics to be used as a drug delivery vehicle for targeted and selective distribution of drugs in various health conditions. In this view, although various studies have been performed *in vivo*, some difficulties have been found, as discussed below in Section 5, and further studies need to be performed.

In a similar manner to GNPs, protein crystals have also been recently recognized as potential carriers of biopharmaceutical drugs for phototherapy applications. The review by Zhou et al. discusses the *in vitro* and *in vivo* crystallization of protein crystals, as well as their biocompatibility and biodegradability, stability, or sustained drug release [6]. Protein crystals, which are characterized by high purity, high stability and a porous structure for biopharmaceutical drug encapsulation, provide a potential avenue with significant advantages for drug delivery and tumor phototherapy. Since fluorescence spectroscopy and imaging have been the most used optical techniques to monitor the delivery of protein crystals in tissues, some recent studies on the use of Hen egg white lysozyme crystals doped with luminescent lanthanide complexes for optical drug sensing *via* fluorescence quenching are described in this review.

The review by Luo et al. describes the use of surface-enhanced Raman spectroscopy (SERS) to monitor kinetic processes of drugs and metabolites in tissues [7]. Common Raman spectroscopy is associated with limited sensitivity, and to overcome this limitation, the mechanisms of electromagnetic (EM) enhancement and chemical enhancement (CE) are used to improve sensitivity of the Raman scattering signals. As referred by the authors, the enhancement factor in the SERS method can be up to 10^9^ through the EM method and up to 10^2^ through the CE method, allowing single-molecule sensitivity. In practical applications, the combination of the EM and CE methods helps to maximize SERS performance. Studies described in this review show that SERS facilitates the identification and quantification of small molecules in biological samples, such as serum, urine, and living cells. Considering the scenarios of drugs and metabolites detection, which have a great variety of applications, keeping accurate track, with good accuracy, of small molecules as they diffuse within biological tissues is not an easy task. The diverse properties of small molecules cause obstacles to accurate and sensitive detection, but since SERS yields a fingerprint spectrum derived from the intrinsic characteristics of molecules, it brings benefits over alternative techniques. SERS also has associated challenges, which will be described in Section 5.

In current Biophotonics there are various optical imaging modalities that are used for various research applications [8]. Optical Coherence Tomography (OCT) is one of these imaging methods that has great applicability to monitor the mobility and delivery in tissues, as described in the paper by Svenskaya et al. [3]. According to these authors, when certain formulations with higher refractive index (RI) than the skin (1.4) are delivered through the HF routes, OCT provides real-time images of the formulation delivery. OCT images showed that prior to the introduction of particles with high RI in the HFs, the HFs appear as dark inclined structures due to their less scattering than the surrounding tissue. After filling the HFs with particles with high RI, such as 120-nm silica cores covered with a 15-nm-thick gold shell and PEG, the HFs become visible as bright white channels due to the increase of scattering. Due to such sensitivity of OCT imaging to the RI changes within the HFs in the skin, such technique can be used to monitor the delivery and absorption of drugs by cells, as discussed in this review. Various fluorescence imaging methods are also discussed to monitor the delivery of particles through the HFs. Using a non-invasive approach like OCT, fluorescence imaging techniques present high resolution, specificity and sensitivity since they allow *in vivo* optical sectioning of the skin to visualize the drug delivery and deposition. For *in vivo* quantitative monitoring of drug delivery through the skin, a promising direction is the integration of the most effective spectroscopy (RS, ERP) and imaging (OCT, confocal laser scanning microscopy (CLSM), two-photon microscopy (TPM), fluorescence lifetime imaging microscopy (FLIM)) methods into multimodal technologies.

Considering still OCT, the review by Aglyamov and Larin, discusses the noninvasive monitoring of drug delivery in many therapeutic procedures [9]. These authors have focused on the latest OCT applications, including OCT-guided drug injection, topical drug delivery monitoring, application of OCT in inhaled drug delivery systems, and the integration of OCT with other imaging modalities, where the advantages of OCT over traditional imaging modalities in terms of spatial resolution, depth penetration, and real-time capabilities are described. The authors refer that recent clinical research demonstrates that OCT can actively assist surgeons during drug injections into delicate tissues, since OCT-guided delivery has the potential to improve both the safety of surgical procedures and the assessment of their outcomes. Although OCT presents various benefits for clinical procedures, some challenges related to the drug delivery monitoring need to be addressed, as discussed in Section 5.

The review by Alexandrovskaya et al. discusses the use of optical coherence elastography (OCE) to quantitatively visualize the kinetics of osmotic strains in tissues due to diffusive penetration of various osmotically active solutions [10]. The magnitude of osmotic strains may range from fractions of 1 % to tens per cent, and the osmotic dehydration and diffusional penetration of active solutes can be obtained from the visualized spatiotemporal dynamics of the strains. The authors indicate that the main features of the OCE-visualized diffusion-front dynamics agree well with Fick’s theory yielding diffusion coefficients that are consistent with literature data. Due to the OCE advantages, this technique may be used to study the diffusion of a broad variety of osmotically-active substances, such as OCAs, cosmetics, drugs, or cryo-preservative agents. This review presents a detailed description of the OCE technology and discusses the latest results and experiments with some OCAs, such as glycerol, diffusing in some tissues like cartilage. The results obtained in these studies show the great potential of OCE for monitoring the mobility of agents in tissues. The OCE approach can be applied *in vivo*, since it needs only one tissue interface to produce measurements. The main challenge presented by the authors is that when the molecular size of the solute is similar to the molecular size of water, the sensitivity of the system to the in-ward flow of the solute and to the out-ward flow of water may not be distinguishable.

The paper from Limcharoen et al. provides a comprehensive analysis of optical imaging techniques used to study microneedle (MN)-facilitated transdermal drug delivery, and highlights the role of optical methods in characterizing MN interactions with the skin, assessing drug delivery, and evaluating treatment efficacy [11]. In this review, authors discuss a broad collection of imaging modalities that have been used to monitor drug delivery to the skin *via* MN, namely: histology, dermascopy, scanning electron microscopy (SEM), fluorescence microscopy, CLSM, OCT, TPM, FLIM, photoacoustic microscopy and laser speckle contrast imaging, or even RS. These methods enable real-time visualization of MN penetration, drug diffusion, and skin response in both preclinical and clinical studies.

Regarding the drug delivery with GNPs, Dykman et al. indicate in their review that optical imaging of release by the GNPs and subsequent biodistribution *in vitro* and *in vivo* is vital for accurate location of affected tissues, to prevent misdosing and improve therapeutic efficacy [5]. Imaging techniques, such as fluorescence imaging, dark-field optical microscopy, magnetic resonance tomography (MRT) and near-infrared (NIR) fluorescence imaging are presented by the authors as the most commonly used to monitor drug release from GNPs and the following biodistribution, being some of these techniques more sensitive to the GNPs as a drug delivery platform.

The research related to drug delivery using nanoparticles (NPs) is a field with great interest in Biophotonics. The review by Belyaev et al. discusses the latest research related to NP drug delivery to tumors using intravital optical microscopy (IVOM) [12]. The delivery of NPs to solid tumors has long relied on the enhanced permeability and retention (EPR) effect, involving permeation of NPs through a leaky vasculature with prolonged retention by reduced lymphatic drainage in tumor. According to these authors, considering recent research studies and clinical data that reveals alternative pathways and approaches of NP delivery, the EPR concept is challenged. The recent application of IVOM revealed delivery mechanisms at cellular level *in vivo*. This review describes these new approaches for drug delivery, including the targeting of tumor endothelium by NPs with subsequent intravascular drug release and gradient-driven drug transport to tumor interstitium, or the exploitation of various immune cells for tumor infiltration and breaking endothelial barriers. With these new discoveries, new challenges have emerged for the drug delivery to tumors *via* NPS. Such challenges and new research direction in this field will be presented in Section 5.

The review by *Banstola* et al. is also related to the subject of using NPs as drug carriers [13]. This paper exploits the latest developments in using alternative energy sources to activate various photosensors, which are delivered by NPs, for achieving high sensitivity, wavelength versatility, and spatial resolution for deep-tissue imaging. The authors describe that unlike the traditional photosensor activation with visible light, other sources, such as NIR, X-rays, and ultrasound can be used for deeper-tissue stimulation of various photosensors. To avoid some challenges, such as tissue auto-fluorescence, in real-time fluorescence imaging, afterglow luminescent NPs are being developed by integrating these alternative energy sources for real-time imaging and sensing in deep tissue for precise cancer diagnosis and treatment beyond superficial tissues. In addition to deep tissue imaging, light-responsive nanomedicines are revolutionizing anticancer and antimicrobial phototherapy by enabling spatially and temporally controlled drug release. These novel NPs are engineered to release therapeutic cargo at target sites in response to microenvironmental signals that are specific to tumors or infections. The controlled drug release by these NPs is made in anticancer phototherapy *via* photoisomerization, photothermal, and photodynamic processes, where the circulation time and specific targeting enhancement can be made with biomimetic NPs that mimic natural anti-tumor responses by the body. In antimicrobial phototherapy, research has been focused on the chemical modification of the photosensitizer to enable targeted drug delivery. The recent development of “pro-photosensitizers”, which are specifically activated within bacterial cells upon light irradiation, offer a high margin of safety. As referred by the authors, these advancements leverage photochemical reactions and nanotechnology to enhance precision diagnostics and therapy in addressing critical health challenges.

The review by Kolesova et al. explores the transformative role of CRISPR/Cas systems in optical bioimaging [14]. CRISPR is the abbreviation for Clustered Regularly Interspaced Short Palindromic Repeats and Cas is the abbreviation for CRISPR-associated protein, enzymes that work with CRISPR sequences to cut DNA. The recent advancements in NP technologies that are revolutionizing the visualization of gene-editing processes both *in vitro* and *in vivo* are emphasized in this paper. A great benefit was provided by the integration of nanoformulated contrast agents in imaging techniques, such as NIR and fluorescence imaging, resulting in improved resolution, sensitivity and specificity on the resulting images. Originally developed for gene-editing, the CRISPR/Cas systems are now being combined with these imaging modalities to enable real-time monitoring and quantitative measuring metabolites, proteins, vitamins, nucleic acids and other entities in specific areas of the body, as well as tracking of CRISPR/Cas delivery, editing efficiency, and potential off-target effects. Enhanced imaging and precise monitoring across multiple scales with multiplexed and multicolor imaging in complex settings, including potential *in vivo* diagnosis became possible with the development of CRISPR/Cas-loaded NPs. With the emergence of biomimetic NPs as promising tools for improving biocompatibility, enhancing targeting capabilities, and overcoming biological barriers, more efficient delivery and bioimaging of CRISPR/Cas systems *in vivo* become more facilitated. As the design of these NPs and delivery mechanisms improves, alongside advancements in endolysosomal escape, CRISPR/Cas-based bioimaging will continue to advance, offering unprecedented possibilities in precision medicine and theranostic applications.

The review by Raju et al. [15], addresses the use of Light-Sheet Fluorescence microscopy (LSFM) to study drug delivery and the development of embryos. Such review highlights the advantages of this method over other imaging techniques regarding image resolution and phototoxicity minimization. LSFM offers 3D images of living organisms, allowing the study of dynamic biological processes. With the minimization of phototoxicity, this technique is ideal for sensitive samples, like moving embryos. Its capability to capture long-term dynamical processes with single cell resolution revolutionized nanomedicine. The new advances in labelled fluorescence imaging and its combination with artificial intelligence (AI) methods have enabled more precise and real-time monitoring of drug release, distribution, and interaction with developing tissues. The LSFM technique provides high speed image acquisition with good spatial resolution, which enables real-time monitoring of drug delivery and expose heterogeneous drug penetration in different locations of complex structures such as tumors. Regarding the research in embryo development, LSFM has also played a crucial role, since it allowed scientists to track organ formation, cell migration, and gene expression during the earliest stages of life.

Kostyusheva et al. address exosomes in their review, as promising tools for targeted drug delivery in biomedical applications and medicine [16]. Exosomes are particles released from cells, with no autoreplication ability, delimited by a lipid bilayer, having endosomal origin and with sizes between 40 and 200 nm. In this review, the strategies for optimizing the synthetic yield and the loading of exosomes with various therapeutics are discussed. The authors describe the various imaging techniques that are used to track exosomes, presenting the ones that are optical-based, such as fluorescent and bioluminescent imaging, and the radiological-based, such as magnetic resonance, radionuclide and single-photon emission computed tomography (SPECT) imaging. During the description of such imaging techniques, some examples of exosome tracking and imaging of their distribution in the liver, lung, brain and other organs are presented.

The use of FLIM for drug delivery research is discussed in the review by Lu et al. [17]. Due mainly to increasingly expanding lifetime based fluorescent sensors for various parameters, FLIM offers specificity and complementary information over conventional fluorescence imaging methods, allowing better discrimination and interpretation of the spatiotemporal distribution, dynamic interaction, and micro-environmental effects of drugs. FLIM is already in a translational stage from the lab to the clinic, with application both in diagnostics and treatment monitoring, sometimes even during fluorescence-guided operation. The authors describe various drug delivery monitoring applications of FLIM, such as in cardiovascular and brain diseases or cancer. Taking advantage of the continuous advances in probe design, which include emerging nanoparticles and composites, in conjunction with instrumentation and algorithms offering improved performance and/or new modalities, FLIM has progressed greatly in the last few years and may improve even further in the near future.

Ozdemir et al. [18] reviewed recent advances in using antimicrobial blue light (aBL; 405–470 nm wavelength), an innovative non-antibiotic and light-mediated approach, to eliminate microorganisms, particularly antibiotic-resistant strains. The authors provided an in-depth analysis of aBL’s mechanisms of action, emphasizing its unique reactive oxygen species (ROS)-driven damaging effects on microbial organelles, such as membranes, DNA, and proteins. Additionally, the authors explored the synergistic combination of aBL with other approaches, including antibiotics, nanoparticles, and natural compounds, for potential enhanced efficacy. They also examined the safety and regulatory considerations associated with aBL treatment.

In the review by Oliveira et al. the use of optical spectroscopy was discussed as a valuable technique to estimate the diffusion properties of OCAs in tissues, to evaluate the mobile water content and discriminate between healthy and diseased tissues [2]. Furthermore, such technique can be used for other clinical applications, such as the study and characterization of the diffusion properties of poisons and their antidotes in various tissues. Obtaining such data will allow the optimization of treatment procedures in the case of tissue poisoning. In the fields of low-temperature organ preservation and food industry, the optimal dosage of the cryopreservation agents, such as glycerol or dimethyl sulfoxide (DMSO), can be calculated from the evaluated diffusion properties of those agents in the tissues to be preserved. Such studies need to be performed to obtain data that will be most useful to optimize the preservation of organs and tissues that will be later used in patients, and also for long-term food preservation at low temperatures. Also in the food industry, the kinetic spectroscopy method can be useful for the controlling the dosage of sugars or preserves to some foods, vegetables and fruits. A wide range of studies with this method is required.

Regarding the EPR techniques discussed in the paper by Svenskaya et al. [3], there is still a reduced number of publications on its use for monitoring particle delivery through the HF routes in the skin. The reason is that EPR spectroscopy for *in vivo* biomedical applications presents limited sensitivity, which is attributed to the hardware/software used to acquire spectra. Such limitation should be addressed soon, so that EPR spectroscopy can be improved and added to the arsenal of optical methods to monitor intrafollicular penetration depth, storage, degradation/metabolization profiles of drug carriers and the release kinetics of drugs they contain.

Considering the view of the various optical clearing applications, the paper from Yu et al. points out the current challenges, such as the uneven distribution of OCAs in tissues, which can result in incomplete clearing of deeper tissue layers [4]. In face of such challenges, the authors propose the future research and development directions, which include the automation of tissue clearing processes that will significantly reduce manual labor and increase efficiency, making it feasible to handle large-scale studies and clinical diagnostics. One other important direction of research indicated by these authors is the potential use of AI to aid the monitoring of the kinetics and delivery of numerous OCAs in a variety of biological tissues.

For the research related to the use of GNPs as drug carriers, the paper by Dykman et al. also indicates the future challenges that should be addressed to translate laboratory experiments into clinical applications [5]. Such application of GNPs *in vivo* should be approached with careful, since there is the possibility of the development of antibodies in human and animal bodies to the administered drug substance adsorbed on nanoparticles, as well as the risk of developing allergic reactions. To perfectly study these subjects, authors recommend the need for *in vivo* studies under controlled conditions. Further studies with different shaped GNPs are also necessary, since up to the present, mainly spherical or quasi-spherical GNPs have been used. Low sorption capacity of gold colloid has been encountered, meaning that GNPs can only be effectively used as a carrier of potent drugs with a therapeutic dose on a microgram scale. This means that to increase the drug loading, one has to fabricate gold-dielectric nanocomposites with available free volumes for loading and release. By adequately modifying the particle surface, the observed drug biodistribution *in vitro* can be efficiently obtained *in vivo*. Renal cleared nanocarriers (RCNC) are referred by the authors as an example of recently emerging drug delivery systems, which enable the drug nanocarrier to quickly penetrate the tumor core without needing prolonged blood retention and avoid uptake by macrophages. Additionally, RCNC can enhance the body’s elimination of non-targeted anticancer drugs, which may be used to improve the therapeutic efficacy of anticancer drug delivery systems and reduce unwanted side effects from their use.

Similarly, when considering protein crystals as drug carriers, some important challenges are presented by Zhou et al. in their review [6]. The phagocyte-mediated clearance, due to the large size of protein crystals, is one of such challenges. Current virus-based protein crystals and bacterial-based Cry3Aa crystals typically range between 400 and 1000 nm in size, which predisposes them to clearance from circulation by phagocytes in the reticuloendothelial system, leading to degradation and loss of therapeutic efficacy following systemic administration. Some measures are proposed to minimize phagocytes clearance and increase therapeutic efficacy. The encapsulation of bigger-sized drugs is currently challenging, since the fixed solvent channels of protein crystals may discriminate against biopharmaceutical drugs based on molecular size. To overcome such difficulty, the authors propose to expand pores *via* mutagenesis, since pore size in protein crystals can be extended by deleting the amino acid residues located on the intermolecular contact region. The uncontrolled release of drugs should also be addressed, since drug release relies on the gradual breakdown of protein crystals under protease conditions. Based on recent studies, the authors suggest that by engineering *in vivo* crystals by mutagenesis will enable an optimized dissolution of protein crystal at lower pH for a better control of the release of encapsulated proteins, which can be stimulated by light or ultrasound. The immunogenicity of the protein crystals also requires further attention, since delivery systems with high immunogenicity can provoke strong immune responses, which will diminish the therapeutic efficacy and potentially cause severe adverse effects. To address this issue, authors point out that systemic investigations are required to further access the immunogenicity of protein crystals, and suggest strategies for minimizing their immunogenicity, such as removing antigen epitopes.

When discussing drug delivery to the skin through microneedles, Limcharoen et al. explore in their review the combination of optical imaging with chemical sensing techniques, which represents a promising direction for future research [11]. These authors emphasize the importance of bridging preclinical and clinical studies through advanced imaging techniques. Other key challenges, such as resolution and imaging depth limitations, are addressed by these authors, alongside future prospects for improving optical visualization of drug delivery applications with microneedles. Enhancing imaging quality and developing multimodal imaging approaches are crucial steps for advancing next-generation microneedle-based drug delivery systems. These authors received new information of other studies that were published at a late date to be included in their review [19–22]. These studies collectively demonstrate the broad applications of optical techniques in microneedle research, ranging from the study of microneedle morphology, *ex vivo* penetration analysis and diffusion studies, intradermal drug distribution to clinical and functional applications of microneedle systems.

The new discoveries provided by the use of IVOM to monitor drug delivery to tumors *via* NPs bring new insights related to the pathways and mechanisms of NP mobility, drug delivery and absorption by tumors. As described in the paper by Belyaev et al. [12], among the most significant discoveries are the dynamic nature of the EPR effect, the involvement of immune blood cells in NP transport, and the recognition of active transcytosis of NPs *via* special nanoparticle transport endothelial cells (N-TECs). These findings were only observed in animal xenograft models, which do not have the exact architecture of human tumors, and molecular xenografts exhibit low heterogeneity and limited genetic diversity. The authors suggest that using patient-derived xenografts or tumors grown from patient-derived cells could improve the relevance of pre-clinical studies. The application of IVOM could benefit from the development of heterogeneous tumors to access the accuracy of these models, where the use of advanced fluorescent cellular sensors for measuring acidity (pH), oxygenation (pO_2_), solid stress and other parameters can precisely access tissue heterogeneity. Since the accumulation of EPR-designed nanomaterials in clinic was highly heterogeneous both in different regions of tumor and between patients, the heterogeneity in newly routes of active NP transport needs to be investigated. The immune cell infiltration levels have been observed to vary significantly among patients with the same tumor type, as well as an unevenly distribution of endothelial cells capable for transcytosis of NPs along the tumor vasculature. These observations turn crucial the necessity of evaluating the contributions of different mechanisms to the overall transport of NPs and to develop the capability to predict tumor response to various treatment options. Selective tracers to target active NP transport mechanisms should also be developed. Additionally, due to the size limit of the endocytic vesicles, a small NP size below 100 nm is beneficial for both the EPR concept and for delivery *via* clathrin- or caveolin-mediated transcytosis. Nanoparticles with larger sizes may exhibit better margination near tumor vasculature and greater interaction with blood immune cells, facilitating chemotaxis-mediated delivery. This means that the optimal size of NPs should depend on the relative contributions of different pathways involved in the NP transport. The investigation on how other physicochemical properties of NPs, such as shape, rigidity, and functional modifications, influence the efficiency of different delivery mechanisms in tumors is also necessary.

Also in the review by Banstola et al. [13], some additional challenges related to NP carriers are presented. These authors indicate that further enhancements in targeting specific tissues or organs, multiplex sensing, and imaging are key areas for future research for developing next-generation photo-sensing technologies. One of the major concerns is the toxicity associated with light-actuated nanomedicine, since light-triggered burst release of therapeutic payloads can result in acute toxicity, and various photosensitive inorganic nanomaterials exhibit poor clearance and systemic toxicity. The complexity of these inorganic nanomaterials represents another significant challenge. A biocompatible, non-toxic and potent nanocarrier that encapsulates diverse photosensitizers and therapeutic agents is desirable, but particularly demanding. Furthermore, the large-scale manufacturing of complex nanosystems for use in larger animal models remains scarce, and tumor heterogeneity is still a major challenge to the therapeutic efficacy of light-activated NPs. The development of NPs with biocompatible materials to reduce toxicity is essential to overcome these challenges. Tumor heterogeneity can also be tackled through personalized nanomedicine approaches and multifunctional nanosystems tailored to individual patient profiles. Regarding antimicrobial phototherapy, these challenges are also applicable, but one promising approach to address selectivity is adopting a pro-drug strategy.

Regarding the CRISPR/Cas systems that are discussed in the paper by Kolesova et al. [14], the lack of efficient and targeted delivery tools is a current challenge that delays the use of this method for diagnostic and therapeutic procedures. The field of CRISPR/Cas delivery technologies is evolving rapidly, but the full potential of these systems for diagnostic and therapeutic applications can only be reached after addressing and overcoming some significant challenges, such as optimizing drug delivery strategies. Regarding this issue, recent advancements in NP design can improve biocompatibility, reduce toxicity, and ensure efficient cellular uptake. Another critical obstacle to ensure that CRISPR/Cas components reach their intended targets within the cell is the achieving of efficient endolysomal escape. Additionally, refining targeting mechanisms to improve biodistribution, minimize off-target effects, and ensure precise delivery to specific tissues and cells will be essential for the success of CRISPR/Cas-based therapies. A crucial aspect that can significantly enhance delivery efficiency is the surface alteration of NPs. Functionalization not only improve the delivery of CRISPR/Cas but also provide a versatile platform for integrating imaging modalities, which can offer insights into the delivery, editing and visualization processes. Overcoming these challenges, joined with the use of novel imaging modalities that provide critical insights into the mechanisms of action, will push CRISPR/Cas-based diagnostics and therapeutics to new heights. According to the authors, these advancements will pave the way for the development of innovative theranostic modalities, making a transformative impact on precision medicine and expanding the potential of gene-editing technologies in clinical applications.

According to Raju et al. [15], although LSFM presents major advantages in the fields of drug delivery and embryo development, as previously mentioned, it also presents some limitations, which translate into some challenges that should be addressed. One such limitation is the fluorophore degradation when exposed to high intensity light for a long time. Tissue opacity and data management are also some limitations associated to this method, which constrain its broader application. Addressing these limitations is critical for the advancement of research in this field. Recent studies have reported that the development of photostable fluorophores and the use of NIR dyes have proven effective in reducing photobleaching and maintaining signal intensity for longer times. Further studies should be conducted to optimize processes of LSFM monitoring of drug delivery and embryo development. Optical clearing protocols, such as CLARITY or CUBIC, have been developed to improve light penetration for image acquisition, and they can help in the LSFM procedures for monitoring drug delivery and embryo development. The combination of improved machine learning algorithms can also help in the data management process and faster recreation of 3D images.

The antimicrobial photodynamic therapy (aPDT) is recognized as a viable treatment strategy for infections resistant to conventional antibiotics. Its use and strategy for photosensitizer and light delivery in aPDT for lung infections is discussed in the review by Shleeva et al. [23]. These authors refer that there is a significant scarcity of studies investigating the efficacy of aPDT in treating lung infections in animal models and also that new optical strategies, such as the application of OCAs, are required for deep lung tissue illumination in pulmonary diseases. According to this review, valuable insights into the mechanisms of aPDT were provided by *in vitro* studies, but to understand the *in vivo* effectiveness, safety, and optimal treatment parameters requires a robust preclinical and clinical investigation. Animal models that mimic human lung infections are essential for the evaluation of the aPDT efficacy in a complex living system, addressing factors such as tissue penetration, immune response, and potential side effects. Well-designed clinical trials to access the safety and efficacy of aPDT in human patients with lung infections are also required before moving from preclinical findings into clinical practice. Although rapid advancements in bronchoscopy technologies and increased clinical expertise are paving the way for the safe and minimally invasive use of aPDT in treating bacterial lung disease, further research is required to develop novel and more efficient light delivery systems that can overcome the challenges of light penetration in the lung. To maximize therapeutic efficacy and minimize side effects, new improvements in wavelength and light intensity optimization, and light exposure time are necessary. The combination of light delivery setup with advanced imaging techniques will enable more precise targeting and monitoring of aPDT treatment. For the successful treatment of lung infections, the understanding and modulation of the immune response to pathogens during aPDT need to be implemented.

Considering the use of FLIM for monitoring drug mobility and delivery, as discussed in the paper by Lu et al. [17], there are some current challenges that should be addressed. Similarly to all fluorescence imaging techniques, brighter and photostable emission is always desirable, where the interdependency between lifetime and intensity plays the fundamental challenge. In various situations, the change in lifetime is associated with reduced intensity, but the lifetime engineering and emission enhancement that have been concurrently achieved in some occasions, especially in the case of nanoparticles, deserve more exploration. The authors point out that lifetime measurement is much more complex than intensity measurement. Due to this challenge, sufficient care should be taken to educate the end-users to avoid experimental artifacts, which is the key to meaningful, reliable discovery and healthy development of the multidisciplinary community. Fluorescence and phosphorence lifetimes have critical dependence on the time scale. Such fact needs to be recognized and taken into account, so that suitable methods and procedures are selected in terms of instrumentation, data acquisition, and lifetime calculation algorithms to ensure accuracy and efficiency, while minimizing misinterpretation. In this context, additional investigation regarding the precise mechanisms underlying lifetime changes is required for a better understanding of the results. Another important challenge is that fluorescent molecules conjugated with a drug may change the actual pharmacokinetics of the drug, as observed in some cases reported in this paper. FLIm has already been integrated with other imaging modalities, such as endoscopy and ophthalmoscopy for complementary and cross-validated information. The addition of machine learning algorithms may benefit various aspects of such integration, such as lifetime estimation, or image processing and analysis, allowing to move forward to real-time and precise diagnostics, which are currently difficult to obtain.

Although SERS yields a fingerprint spectrum derived from the intrinsic characteristics of molecules, as described by Luo et al. in their paper [7], such fingerprint characteristic has limitations, since only certain groups of molecules produce strong Raman signals and the presence of structurally similar substances and metabolites greatly complicates detection in biological samples. As described by the authors, in order to achieve more accurate and reliable detection, it is necessary to understand as much as possible about how the analyte interacts with plasmonic nanoparticles when establishing the method. In terms of precise quantification in biological samples, it is necessary to perform appropriate pretreatment to enrich the metabolites and reduce the interference of the matrix, a procedure now lacking of standardized protocols in the field of Raman spectroscopy. Regarding the substrates used with SERS, excellent uniformity is exhibited by etched substrates but they are costly, while suspended colloidal substrates are easy to synthesize, yet controlling their shape and size is challenging. This means that the development of new affordable, easily mass-producible substrates is crucial. On the other hand, the authors refer that although many studies have reported the use of SERS for the detection of disease biomarkers and drugs, often with outstanding detection limits, the reproducibility of these results across different laboratories is still challenging. Before translating SERS technology to the clinical environment, it is crucial to conduct cross-laboratory and multi-center validations to reach clinical-grade precision. Finally, the use of AI is one possible tool to help SERS researchers to overcome some current challenges such as signal background removal, spectral noise reduction, molecular identification and disease diagnosis.

OCT for monitoring drug delivery in various therapeutic applications presents many advantages, as described in the review by Aglyamov and Larin [9]. However, these authors indicate that the application of OCT in monitoring drug delivery remains constrained by limited sensitivity to molecular dynamics. Such limited sensitivity could be improved by incorporating novel functional extensions and advanced contrast agents, elevating OCT to a new diagnostic level. One possible direction to be considered is the integration of OCT into multimodal systems, which could contain fluorescence, microscope, ultrasound or photoacoustic imaging modalities, or even Raman spectroscopy. Such integration of different imaging modalities would allow to capture detailed information about biological and biochemical processes within tissues. When such challenges are overcome, the imaging and diagnostic capabilities of OCT can be improved, turning this technology into an effective tool for evaluating drug efficiency on short- and long-term scales, allowing to monitor treatment outcomes locally and optimize novel treatment strategies.

For using aBL to treat microbial infections, Ozdemir et al. [18] noted that attention must now shift toward overcoming existing limitations and integrating emerging technologies to support its broader application. They highlighted the integration of AI-driven analytics and sensor-guided light delivery systems. Additionally, the authors underscored the pressing need for standardized aBL treatment protocols, specifying wavelength, dose, and treatment duration, as the lack of such standards remains a critical barrier to clinical translation.

The use of optical technologies in monitoring the mobility and delivery of drug and metabolic agents is one of the major research lines of Biophotonics at present day. Various spectroscopic and imaging methodologies have been used in this research field, allowing to achieve various results and improvements in the last 20 years. Although most of these results have been obtained from *ex vivo* studies, some *in vivo* and pre-clinical trials have demonstrated the applicability of optical technologies for diagnostic and therapeutic applications where the monitoring of the mobility and delivery of drugs is crucial.

Kinetic collimated transmittance spectroscopy measurements in the visible range provide the adequate sensitivity to monitor the mobility and delivery of OCAs in tissues, allowing to estimate the characteristic diffusion time and diffusion coefficient properties of OCAs in tissues, as well as to evaluate the mobile water content in healthy/diseased tissues. Such methodology can be used *in vivo*, by replacing the collimated transmittance setup by a diffuse reflectance sensor, which is also sensitive to the OCA mobility within the tissues. The translation of this methodology to clinical applications can be made by involving multidisciplinary research teams and patient volunteers, allowing to establish a relationship between the mobile water content and cancer progression for an accurate diagnosis. New studies using this methodology to evaluate the diffusion properties of poisons and their antidotes should be made as soon as possible, so that the establishment of efficient poison treatment protocols can be developed. The application of kinetic spectroscopy measurements in studies to evaluate diffusion properties of cryoprotective agents, such as glycerol or DMSO is immediate, and studies should be made to optimize agent dosage for long-term cryoprotection of organs and cells. In food industry, the evaluation of sugars and other food additives or preservatives can also be studied using this method for optimization of food preservation, sweetness and quality control processes. Although most of these agents are also OCAs, the reversibility of adding them to organs and food product should also be studied.

In the fields of cosmetology and dermatology, the delivery of drugs and agents *via* microneedles or through the HF routes present significant advantages and recent interest in terms of delivery efficiency to the deep layers of the skin. Raman spectroscopy is mostly used in a noninvasive configuration, and significant results have been recently obtained using this spectroscopic modality in monitoring the delivery of drugs and NP in the skin. Along with Raman spectroscopy, FTIR and fluorescence spectroscopy have also been used to monitor de mobility and delivery of NPs, protein crystals and other drug carriers, as well as to evaluate their drug release efficiency and drug biodistribution in various tissues and cells.

In similarity to spectroscopy methods, various imaging techniques, such as OCT, confocal laser scanning microscopy, two-photon microscopy, LSFM have also been used to perform the *in vitro* and *in vivo* monitoring of the mobility and delivery of various drugs, exosomes OCAs, or drug carriers. With the possibility of creating 3D images and real-time monitoring of the pathways of drugs and drug carriers, optical imaging techniques provide a good tool for clinicians to evaluate treatment precision and efficiency.

The research in the fields of optical imaging and spectroscopic modalities to monitor drug mobility and delivery is an ongoing research that has identified various challenges in the latest years. Those challenges are perfectly identified, and the continuous effort in this field of research will certainly overcome them shortly to proceed with the translation of these technologies from the lab to the clinic. One possible solution to improve these methods is the integration of AI to improve image resolution and detection precision, or to help avoiding inaccurate results. Some other potential applications of the use of optical technologies for monitoring the mobility and delivery of agents have already been identified by researchers, but possibly in the following years, other possible applications will emerge, where such technologies can be applied.

## Figures and Tables

**Fig. 1.**
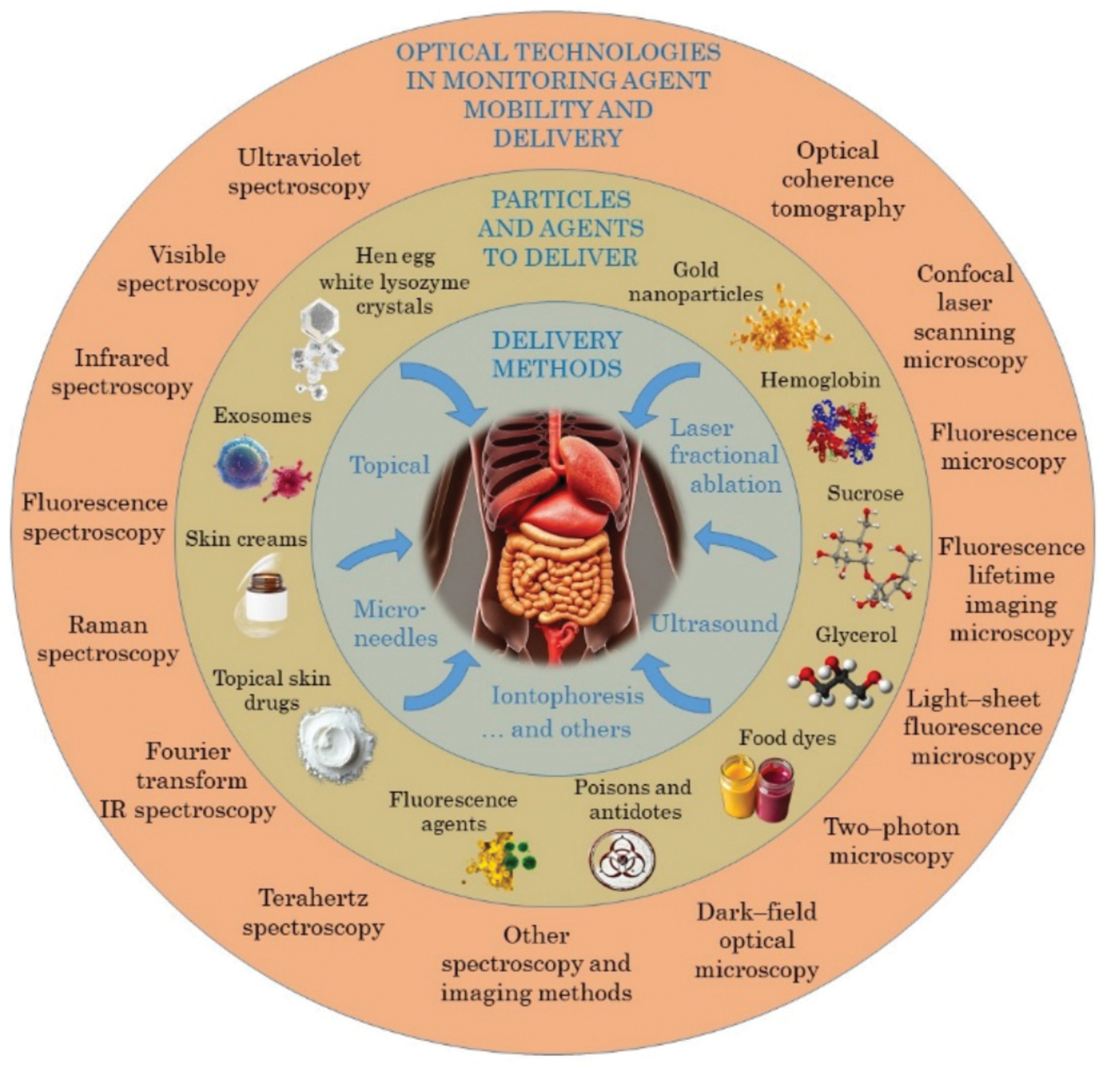
Current optical technologies, typical particles and agents, and delivery methods.

## Data Availability

No data was used for the research described in the article.
